# Experimental Investigation of Unconfined Compression Strength and Microstructure Characteristics of Slag and Fly Ash-Based Geopolymer Stabilized Riverside Soft Soil

**DOI:** 10.3390/polym14020307

**Published:** 2022-01-13

**Authors:** Zhengdong Luo, Biao Luo, Yufei Zhao, Xinyu Li, Yonghua Su, He Huang, Qian Wang

**Affiliations:** 1 College of Civil Engineering and Mechanics, Xiangtan University, Xiangtan 411105, China; luozhengdong0425@163.com (Z.L.); wangqian19970630@163.com (Q.W.); 2 China Institute of Water Resources and Hydropower Research, Beijing 100041, China; 3 Hunan Xihu Construction Group Co., Ltd., Changsha 410031, China; shencp1210@163.com; 4 College of Civil Engineering, Hunan University, Changsha 410082, China; suyonghua1965@163.com; 5 Yueyang City Roads and Bridge Construction Corporation, Yueyang 414002, China; Huanghe6668881@163.com

**Keywords:** geopolymer, riverside soft soil, stabilizer, unconfined compression strength, microstructure characteristics

## Abstract

To solve the issues of insufficient early strength of cement stabilized soil and high resource cost, high reduction cost, and high environmental cost induced by the application of cement, the slag and fly ash-based geopolymer was adopted as the stabilizer to treat riverside soft soil. This study mainly investigated the effects of stabilizer content, slag-to-fly ash ratio, and alkaline activator content on the strength of geopolymer stabilized soils with different curing ages. Unconfined compressive strength (UCS), scanning electron microscope (SEM), and X-ray energy spectrum analysis (EDS) tests were carried out. The results show that the stabilizer content, slag–fly ash ratio, and alkaline activator content have a decisive influence on the UCS of geopolymer-stabilized soil. The mix-proportions scheme of geopolymer stabilized riverside soft soil, with a geopolymer content of 15%, a slag–fly ash ratio of 80:20, and an alkaline activator content of 30%, is considered optimum. It is proven by SEM that the uniformly distributed gelatinous products formed in the geopolymer-stabilized soil bind the soil particles tightly. Moreover, the EDS analysis confirms that the gelatinous products are mainly composed of C-S-H gel and sodium-based aluminosilicate (N-A-S-H).

## 1. Introduction

The riverside soft soil in the Xiangjiang River Basin in China has the physical characteristics of high moisture content, high compressibility, and low strength, and the soil contains a certain amount of organic matter, which can easily cause instability or uneven settlement of the foundation. This poses severe challenges to the construction, operation, and maintenance of infrastructure. Soft-soil solidification technology, as a very widely used soft-soil foundation treatment method, specifically refers to a series of chemical and physical interactions between stabilizers and soft soil to form stabilized soil with sufficient strength [1,2]. At present, Portland cement is the most commonly used stabilizer in foundation treatment, and there are many studies on the mechanical properties and theoretical aspects of cement stabilized soil [3,4,5,6]. In order to significantly improve the engineering characteristics of the existing soil, the content of cement adopted often reaches 15–20% of the soil quality. However, the production process of cement consumes a lot of resources and energy, and emits more greenhouse gases and pollutants, which imposes a huge burden on environmental protection [7]. In addition, cement stabilized soil often has disadvantages such as insufficient early strength and poor durability [8], which affects the quality and construction period of the project. Therefore, it is essential to develop environmentally friendly stabilizers with excellent performance by replacing cement.

Geopolymer is a new type of cementitious material with a three-dimensional network structure prepared from natural or artificial silicon-alumina materials as raw materials through polymerization such as strong alkali action and lattice reconstruction [9,10]. In recent years, scholars from various countries have devoted themselves to the use of low-cost industrial and agricultural solid wastes in the preparation of geopolymers, which can effectively reduce cement consumption [11,12,13,14,15,16]. Moreover, its energy consumption and CO_2_ emissions are approximately 60% and 10–20% of Portland cement [17,18,19,20,21,22,23]. Previous studies have shown that the mechanical properties of slag geopolymers depend on the concentration and modulus of the alkaline activator solution [24,25,26]. Provis [27] pointed out that the highest strength of fly ash-based geopolymer was obtained in the range of the modulus of the alkaline activator from 1.0 to 1.5. Sultan et al. [28,29,30] investigated the influence of various factors such as slag content, alkaline activator content, and water–binder ratio on the mechanical properties of composite geopolymer paste and geopolymer concrete. Zhou [31] used molecular dynamics simulation (MD), scanning electron microscopy (SEM), and X-ray diffraction (XRD) to investigate the Young modulus, shear modulus, phase composition, and micromorphology of slag and fly ash-based geopolymers at different SiO_2_/Al_2_O_3_ ratios. The results showed that the hydration reaction process of geopolymers could be roughly summarized as: the ions generated by the dissolution of the raw materials formed silicon and aluminum gels in the presence of alkaline activators, and further polymerized to form network structures or zeolites.

Recently, geopolymers have been mainly used as structural materials, insulating materials, and repair materials [32,33,34,35,36]. However, the research and application of geopolymers in stabilizing soft soils has been rather limited. In contrast, the use of geopolymers instead of cement as stabilizers for soft-soil foundations can not only effectively solve the problems of cement of “high resource cost, high reduction cost, high environmental cost”, and industrial waste disposal, but also can further enhance the early strength of stabilized soil and reduce construction costs. Abdullah [37] studied the mechanical properties of fly ash-based geopolymer stabilized clay such as shear modulus, excess pore water pressure, and strain response, and confirmed that geopolymer has good potential in treating clay. Arulrajah et al. [38,39,40,41,42,43] found that the stabilizer content, geopolymer ingredients, component quantities, and curing age significantly affected the strength formation of the treated soil. Chen [44] evaluated the compressive strength of slag–fly ash-based geopolymer stabilized soil with different stabilizer content (8–14%), moisture content (30–60%), and curing age (0–28 d). The results show that the strength reaches the maximum value of 527.09 kPa when 14% geopolymer is added. In addition, the synergistic effect of hydrated calcium silicate (C-S-H) and sodium-based aluminosilicate (N-A-S-H) contributes to form a more compact soil microstructure. Abdullah [45] used consolidated undrained triaxial compression and unconfined compressive strength (UCS) tests to study the stress–strain behavior of geopolymer stabilized clay soils in the presence of different geopolymer content (0, 10%, 20%). Meanwhile, it was pointed out that the overall performance differences of stabilized clay soils were mainly due to the heterogeneity of the soil minerals. Although the above-mentioned research is closely related to geopolymer stabilized soil, it has not proposed specific mix-proportions schemes based on the actual engineering background, especially the riverside soft soil as an example. Moreover, considering factors such as stabilizer content, slag–fly ash ratio, alkaline activator content, and curing age, there are few relevant reports on the strength and microstructure characteristics of geopolymer stabilized soils based on slag–fly ash.

In this study, slag and fly ash-based geopolymer was selected to replace cement as a stabilizer for stabilizing riverside soft soil. On the basis of the preliminary experiment, a “three-factor, three-level” orthogonal experiment was designed to investigate the influence of stabilizer content, slag–fly ash ratio, and alkaline activator content on the UCS of stabilized soil at 7 d and 28 d. Meanwhile, a group of cement stabilized soils with the same stabilizer content was set for comparison. Scanning electron microscope (SEM) and X-ray energy spectroscopy (EDS) test methods were used to observe and compare the microstructure evolution of cement/geopolymer stabilized soil.

## 2. Materials and Test Program

### 2.1. Materials

The test soil was obtained from the riverside in the Xiangjiang River Basin in China, which is a typical silty soft clay. Its basic physical characteristics are shown in Table 1. The undisturbed soil samples were dried to constant weight in an oven at 105 °C, then passed through a 5 mm screen, and stored in a sealed bucket.

**Figure 1:**
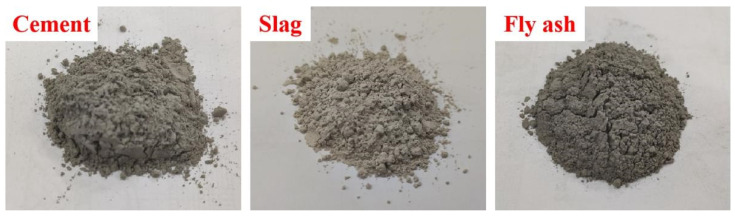
Photos of raw materials.

In this study, the soft-soil stabilizers used included P·O 42.5 cement (Hunan Southern Cement Group Co., Ltd., Changsha, China) and slag and fly ash-based geopolymer. S95-grade slag (Gongyi Longze Water Purification Material Co., Ltd., Zhengzhou, China) and F low-calcium fly ash (Gongyi Yuanheng Water Purification Material Factory, Zhengzhou, China) were used as raw materials of geopolymer, which conform to the Chinese standard GB/T 27690-2011 [43,44]. Figure 1 shows the appearance of cement, slag, and fly ash, and their specific surface areas are 342, 505, and 935 m^2^/kg, respectively. Table 2 represents the composition of raw materials obtained by an X-ray fluorescence (XRF) spectroscopy instrument. The alkaline activator of geopolymer is a mixed solution of NaOH and sodium silicate. The initial modulus of the sodium silicate solution (the ratio of the mass fraction of SiO_2_ to Na_2_O) is 3.31. The composition and properties of the prepared geopolymer paste are shown in Table 3.

### 2.2. Mix Proportions and Sample Preparation

The purpose of this experiment was to clarify whether the stabilizing effect of slag and fly ash-based geopolymer on riverside soft soil is equal to or better than that of cement, and to obtain the optimal mix-proportions scheme. In this study, the remodeled soil with an initial moisture content of 52.0% (the ratio of moisture to dry soil mass) was prepared again. The water–binder ratio of the stabilizer was constant at 0.5. On the basis of the preliminary experiment, a “three-factor, three-level” orthogonal experiment was set up. The contents of cement and geopolymer (the ratio of stabilizer to remodeled soil mass) were 12%, 15%, and 18%, respectively. In addition, in order to study the influence of slag–fly ash ratio and alkaline activator content on the strength of geopolymer stabilized soil, three slag–fly ash ratios (90:10, 80:20, and 70:30) and three alkaline activator contents (20%, 30%, and 40%) were adopted. The specific mix proportions are shown in Table 4.

### 2.3. Sample Preparation

The sodium silicate solution and a certain amount of NaOH flakes were uniformly mixed by magnetic stirring to prepare a sodium silicate solution with a modulus of 1.20 as an alkaline activator. Taking into account the rapid heat release of NaOH in contact with water, the alkaline activator was prepared 1–2 h in advance, and then mixed into the uniformly mixed slag–fly ash dry powder to produce geopolymer pastes.

In order to obtain a uniform soil structure, cement/geopolymer stabilized-soil samples were prepared according to the following procedure: (1) The stabilizer (cement and geopolymer) was prepared according to the above-mentioned mix proportion; (2) The stabilizer paste was continuously and uniformly mixed into the remodeled soil, and stirred until the mixture reached a uniform state; (3) According to the Chinese standard JGJ/T 233-2011 [46], the soil mixture was filled to a cube mold with a size of 70.7 mm × 70.7 mm × 70.7 mm, and vibrated for 1–3 min to eliminate air bubbles in the sample; (4) The surface of the soil sample was covered with plastic wrap to prevent loss of water, and then placed in a curing box with a temperature of 20 ± 2 °C and a humidity range of 95 ± 1% for 1 d; (5) After curing for 1 d, the soil samples were demolded and continued to be sealed with plastic wrap, and then put into the curing box until the test time.

### 2.4. Test Methods

**Figure 2:**
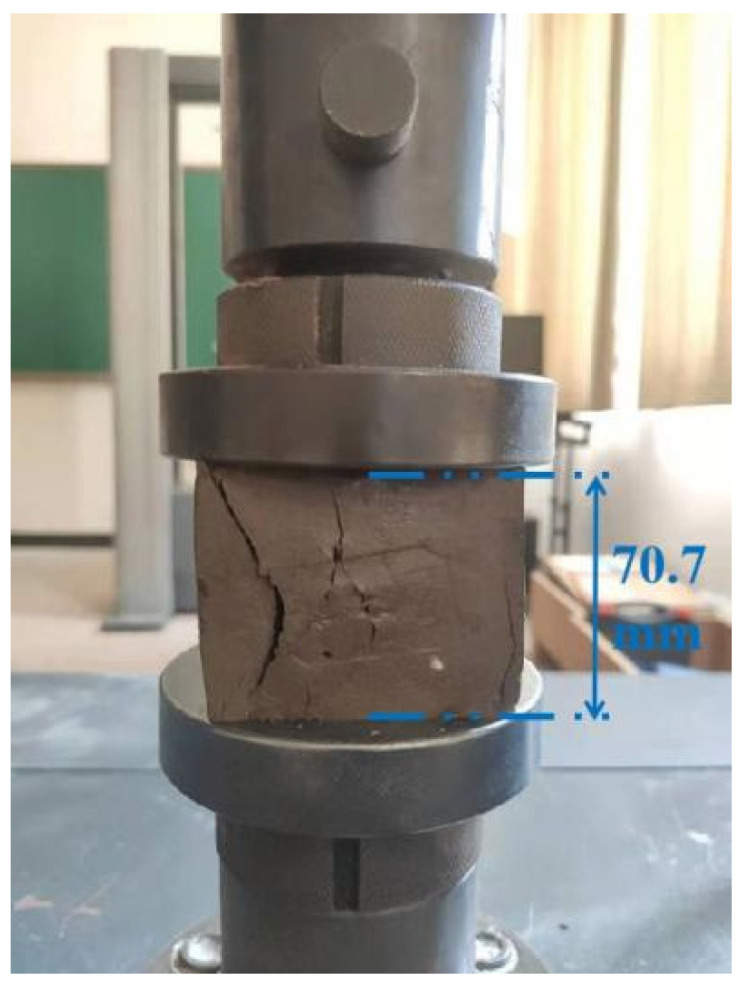
Unconfined compressive strength test.

As shown in Figure 2, according to the Chinese standard JGJ/T 233-2011, the unconfined compressive strength (UCS) of the sample was measured by the CMT5105 electromechanical universal testing machine (MTS Systems (China) Co., Ltd., Shanghai, China) with a maximum force of 100 kN, and the loading rate was set to 1 mm/min. The average value of the strength of the six samples was taken as the representative strength.

The TESCAN MIRA4 type scanning electron microscope (SEM, TESCAN, Ltd., Brno, Czech Republic) and Xplore type X-ray energy spectrometer (EDS, Oxford Instruments (Shanghai) Co., Ltd., Shanghai, China) were used to observe the microstructure and product phases of stabilized-soil samples, revealing the stabilization mechanism that changes with the curing age in the presence of different stabilizers. The accelerating voltage of the SEM used was 0.2–30.0 keV, and the resolution was 2–20 μm. The energy resolution of the EDS was 129 eV@100,000 cps. The tested sample was taken from the inside of the sample damaged in the UCS test. Before the microstructure analysis, the sample should be gold-plated on the section to be observed to prevent the surface from being discharged due to the accumulation of electric charges, so as to avoid affecting the image quality.

## 3. Results and Discussion

### 3.1. Effect of the Type and Content of Stabilizer on the UCS of Stabilized Soil

**Figure 3:**
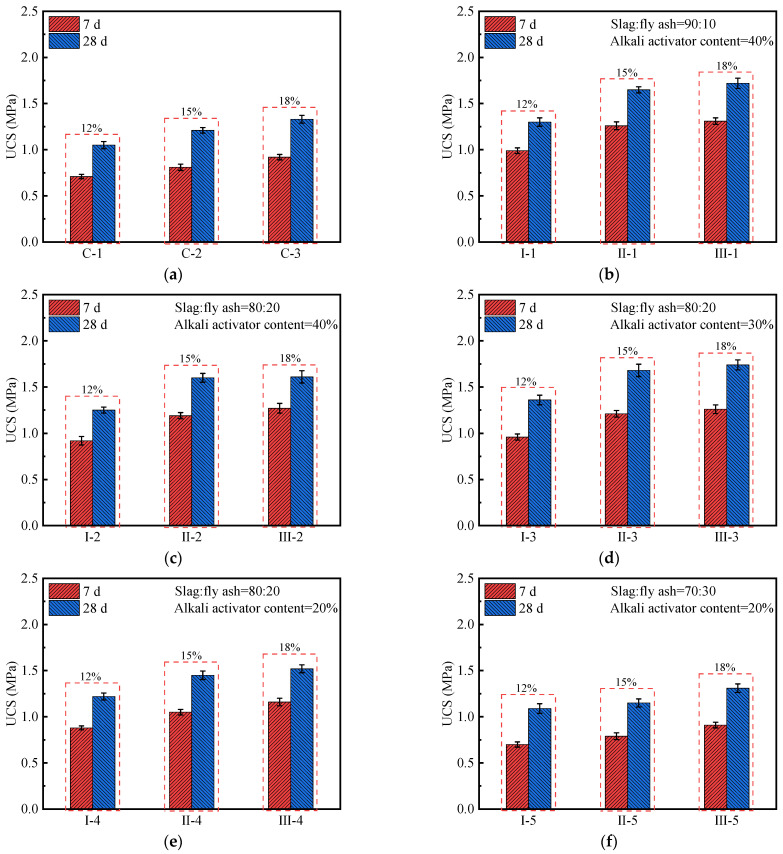
The UCS value of cement/geopolymer stabilized soil with different stabilizer contents (12%, 15%, and 18%): (**a**) Cement stabilized soil; (**b**–**f**) Geopolymer stabilized soil.

Figure 3 shows the effect of different stabilizer content on the strength of cement/geopolymer stabilized soil. On the whole, increasing the stabilizer content and prolonging the curing period can increase the UCS of stabilized soil. Figure 3a shows that, when the stabilizer content is 12%, 15%, and 18%, the 7 d UCS of cement soil are 0.71, 0.81, and 0.92 MPa, respectively, and the 28 d UCS are 1.05, 1.21, and 1.33 MPa, respectively. It can be seen that the UCS of cement soil almost increases linearly with the increase in stabilizer content. This shows that the optimal cement content is above 18%. Meanwhile, it is found that the 7 d to 28 d strength ratio (*ω*) of cement soil ranges from 66.9% to 69.2%.

It can be seen from Figure 3b–f that, for the geopolymer stabilized-soil samples, their UCS increases with the increase in the stabilizer content, but the growth rate gradually decreases. For instance, in the case where the slag:fly ash ratio is 90:10 and the alkaline activator content is 40%, when the stabilizer content is increased from 12% to 18% (I-1, II-1, and III-1), the 28 d UCS of geopolymer stabilized soils are 1.30, 1.65, and 1.72 MPa, respectively. It can be seen that the 28 d UCS of III-1 is 32.3% higher than that of I-1, but only 4.2% higher than that of II-1. This may be due to the presence of some unreacted slag and fly ash particles in the stabilized soil, which leads to the deterioration of the internal microstructure of the sample [38,39,40,41,45,47]. This hinders further improvement in strength. Therefore, from the perspective of strength test results and economy, 15% is the optimal stabilizer content.

Compared with the cement soil, the geopolymer soil has a higher ω value in the case of the same stabilizer [48]. Different from the incomplete hydration of cement in the early stage, the geopolymer reacts rapidly under the action of alkaline activator. Meanwhile, it is found that the strength of the geopolymer soil is always higher than the cement soil at the same cured age. When the stabilizer content is 15%, the 28 d UCS of cement soil (C-2) and geopolymer stabilized soil (II-1) are 1.21 and 1.65 MPa, respectively, which are 40.3 times and 55.0 times that of the undisturbed soil (0.03 MPa). In addition, the strength of II-1 is 1.36 times that of C-2. The results show that cement and geopolymer are used as the stabilizer for stabilizing soft soil, which has significantly improved the mechanical properties of the soil [38,39,40,41,42,43,44,48]. It is worth noting that the geopolymer has a better stabilizing effect on the riverside soft soil, which may be attributed to the pozzolanic reaction in the geopolymer soil [49]. The incorporation of the geopolymer increases the silicon phase and calcium phase in stabilized soil, and the alkaline activator dissolves the amorphous silicon, aluminum, and calcium in the slag and fly ash, forming a large number of monomers. As a result, a polycondensation gel mesh is constructed, which can wrap the soft-soil particles and fill the gaps between the particles, thereby improving the overall strength of stable soil [50,51]. In addition, there are also some silicon phases and amorphous phases in soft soil, which may also contribute to the formation of geopolymer network structure in the presence of an alkaline activator [52].

### 3.2. Effect of the Slag–Fly Ash Ratio on the UCS of Geopolymer Stabilized Soil

**Figure 4:**
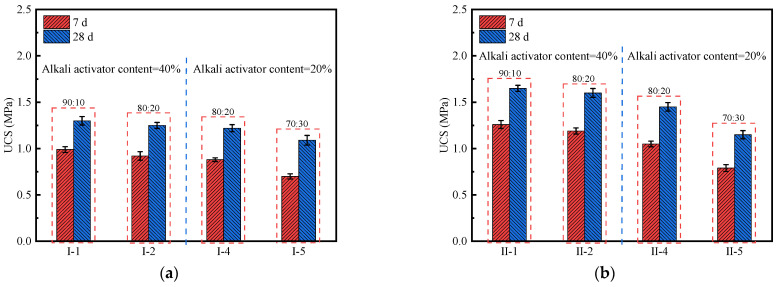

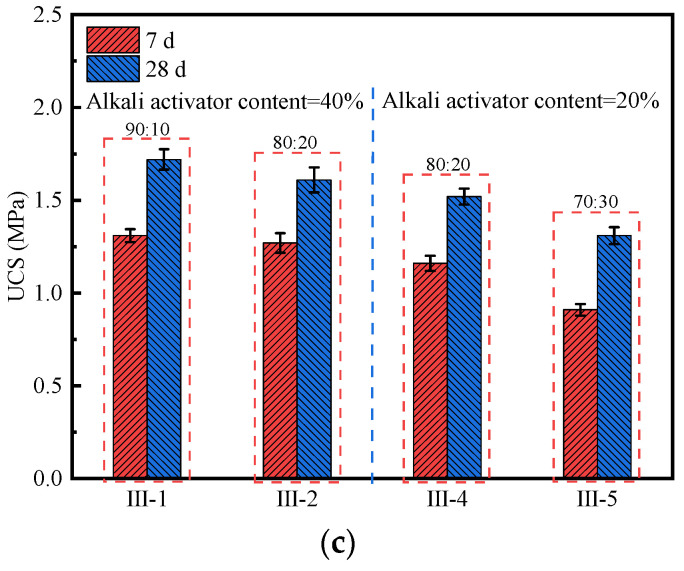
The UCS value of geopolymer stabilized soil with different slag–fly ash ratios (90:10, 80:20, and 70:30): (**a**) Geopolymer content = 12%; (**b**) Geopolymer content = 15%; (**c**) Geopolymer content = 18%.

Figure 4 shows the evolution of UCS of geopolymer stabilized soil with the slag–fly ash ratio and curing age. As shown in Figure 4b, under the condition that the geopolymer content is 15% and the alkaline activator content is 40%, the 28 d UCS of II-1 and II-2 are 1.65 and 1.60 MPa, respectively. Their ω values are 76.4% and 74.4%, respectively. Meanwhile, under the condition that the alkaline activator content is reduced to 20%, the 28 d UCS of II-4 and II-5 are 1.45 and 1.15 MPa, respectively. Their ω values are 72.4% and 68.7%, respectively. A similar evolution law is observed in Figure 4a,c.

It can be seen from the UCS test results in Figure 4 that the strength of the geopolymer soil with a large proportion of slag develops faster and obtains a higher 28 d UCS. For the stabilized-soil samples with high slag content (90%), silicate and aluminate ions are quickly generated in the presence of an alkaline activator. Then, they polymerize with Ca^2+^ ions to form a large number of overlapping gel products, which is beneficial to the strength development of stabilized soil. It can also be seen that the UCS of the geopolymer soil only slightly decreases as the slag–fly ash ratio changes from 90:10 to 80:20. This is mainly due to the incorporation of more spherical fly ash particles, which has a rolling and lubricating effect on the geopolymer paste, which contributes to obtaining a uniform stabilized-soil structure [52]. Meanwhile, in the presence of an alkaline activator, the silicon-rich phase and aluminum-rich phase in fly ash can provide active SiO_2_ and Al_2_O_3_ components for the Ca^2+^ ions separated from the slag, thereby promoting the generation of a large amount of gelatinous products, such as calcium silicoaluminate hydrate (C-(A)-S-H) [52,53], so that the strength of geopolymer soil remains roughly stable. However, the stabilizing effect of geopolymer on soft soils is significantly weakened as the fly ash content increases by 30%. The main reason may be that the Si-O bond and the Al-O bond are first fractured under the action of the alkaline activator, and then form a three-dimensional aluminosilicate network structure (N-A-S-H gel) by the polycondensation reaction, which contains SiO_4_ and AlO_4_ tetrahedral. Such a structure is very stable, which hinders its secondary reaction with the hydrate of the slag, thereby weakening the adhesion of the geopolymer on the soft-soil particles [50,52].

In conclusion, whether the slag–fly ash ratio in geopolymer soil is 90:10 or 80:20, their strength is almost equivalent. Among them, the stabilized soil with the slag–fly ash ratio of 80:20 has better fluidity. In addition, the market price of slag is higher than fly ash. Therefore, it is recommended to adopt 80:20 as a slag–fly ash ratio for geopolymer soil.

### 3.3. Effect of the Alkaline Activator Content on the UCS of Geopolymer Stabilized Soil

**Figure 5:**
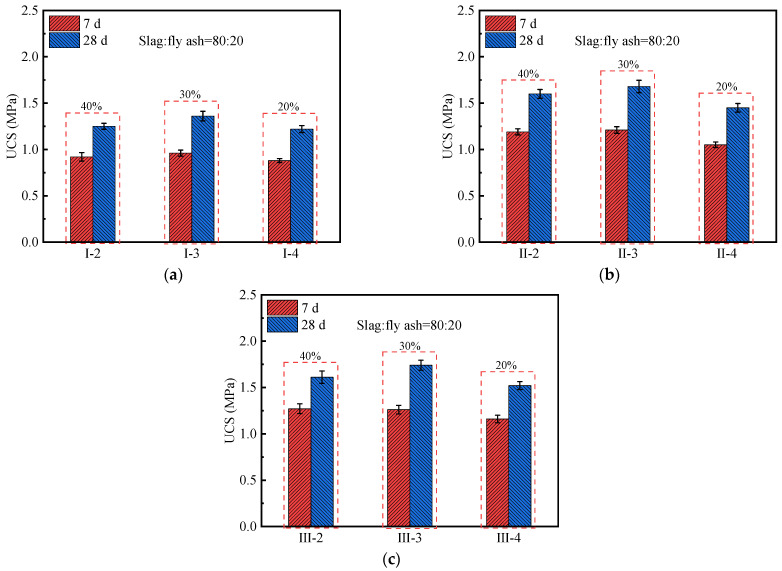
The UCS value of geopolymer stabilized soil with different alkaline activator contents (40%, 30%, and 20%): (**a**) Geopolymer content = 12%; (**b**) Geopolymer content = 15%; (**c**) Geopolymer content = 18%.

Figure 5a–c show the variation of the UCS of geopolymer soil with the alkaline activator content. It can be found that the UCS of the geopolymer soil increases first and then decreases with the increase in the alkaline activator content. The incorporation of the alkaline activator improves the content of OH^−^ and SiO_3_^2−^ ions in geopolymer soil, resulting in the rapid dissociation of the geopolymer precursors and the formation of gelatinous hydration products [50,54]. As shown in Figure 5b, under the condition that the geopolymer content is 15% and the slag–fly ash ratio is 80:20, the 7 d and 28 d UCS of II-4 are 1.05 and 1.45 MPa, respectively. It can be speculated that the low content (20%) of alkaline activator is unable to completely excite the aluminosilicate in slag and fly ash, which limits the development potential of stabilized-soil strength [54]. When the alkaline activator content increases to 30%, the 7 d and 28 d UCS of II-3 are 1.21 and 1.68 MPa, respectively. Its 28 d UCS is 56.0 times that of the undisturbed soil and 1.39 times that of C-2. However, when the alkaline activator content increases from 30% to 40%, it will not further improve the mechanical properties of stabilized soil, and even produce a slight deterioration effect on the UCS. Compared with II-3, the 7 d and 28 d UCS of II-2 decrease by 1.7% and 4.8%, respectively. The higher alkaline activator content results in the rapid release of Al^3+^, Si^4+^, and Ca^2+^ in the geopolymer pastes, thereby effectively reducing its initial setting time. During the sample preparation process, it is found that the geopolymer paste has a solidification phenomenon when mixed with the remodeled soil. This has an adverse effect on the formation of stabilized-soil structure [52]. Therefore, the optimal alkaline activator content is 30% for the geopolymer soil with the slag–fly ash ratio of 80:20.

### 3.4. Microstructure Characteristics of Stabilized Soil

To study the microscopic morphology of stabilized soil at different curing ages, this section details SEM analysis on geopolymer stabilized-soil samples (II-3 and II-5, and their slag–fly ash ratios of 80:20 and 70:30, respectively) with a stabilizer content of 15%, and cement stabilized-soil control group (C-2) is added for comparison. Additionally, based on the SEM image, elemental analysis of the characteristic products is performed with an X-ray energy spectrometer (EDS) to obtain the chemical composition. The highlighted EDS spectrum analysis area has been marked with a white circle, and the obtained relative intensity values of the elements are listed in the table.

**Figure 6:**
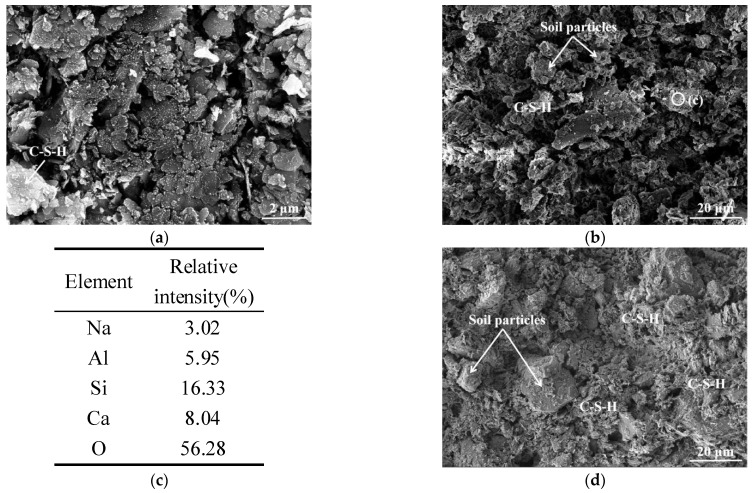
SEM images of C-2: (**a**) and (**b**) 7 d; (**c**) EDS result—7 d; (**d**) 28 d.

Figure 6a,b show SEM images of cement stabilized soils with a size of 2 μm and 20 μm at 7 d. It can be seen that the gelatinous hydration products generated by cement hydration are wrapped on the surface of soil particles, and the voids between the soil particles are not effectively filled, resulting in a relatively large dispersion of soil particles. According to the results of EDS analysis (Figure 6c), the Ca and Si elements in the observed gelatinous products account for 8.04% and 16.33% of the total, respectively, indicating that this type of hydration product is hydrated calcium silicate (C-S-H) [52]. Figure 6d shows the SEM image of the cement soil cured for 28 d. As the hydration reaction continues, the gelatinous products overlap each other, which makes the microstructure denser, thereby increasing the UCS of the stabilized soil. It is worth noting that there are still some soil particles exposed on the surface of the microstructure.

**Figure 7:**
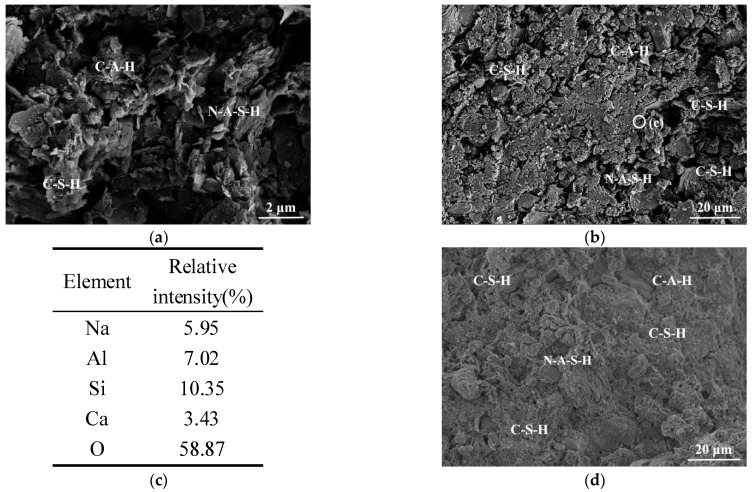
SEM images of II-3: (**a**) and (**b**) 7 d; (**c**) EDS result—7 d; (**d**) 28 d.

It can be seen from Figure 7a,b that the slag and fly ash rapidly hydrate in the presence of the alkaline activator, generating a large number of uniformly distributed gelatinous products which bind the soil particles together. Compared with C-2, II-3 forms a denser microstructure, which is manifested as an increase in the UCS from a macro perspective, as described in Section 3.3. In addition, almost no spherical fly ash particles are observed in the SEM image. This indicates that the activity of fly ash is activated in the alkaline environment generated by the alkaline activator, so that the fly ash particles are gradually covered by the generated hydration products [40,41,42,52]. The EDS analysis result of Figure 7c shows that the geopolymer gel (point c) is composed of hydrated calcium silicate (C-S-H) and sodium-based aluminosilicate (N-A-S-H). The N-A-S-H gel is mainly formed by the “dissolution–polymerization” reaction of active substances such as Si and Al in the alkali–fly ash system [44,50,52]. As shown in Figure 7d, the internal particles of the geopolymer soil sample at 28 d are fully bonded and consolidated to form a whole.

**Figure 8:**
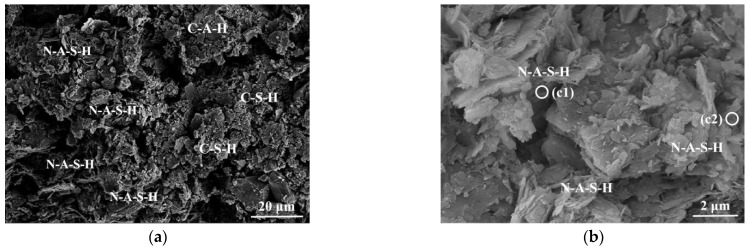

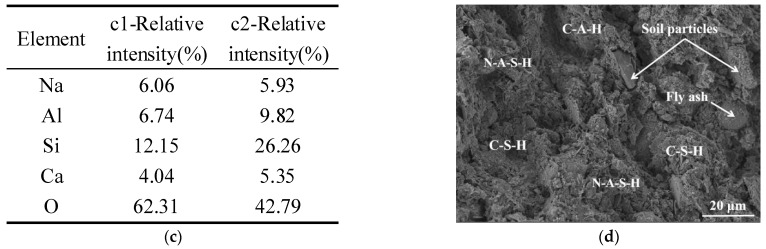
SEM images of II-5: (**a**) and (**b**) 7 d; (**c**) EDS result—7 d; (**d**) 28 d.

As shown in Figure 8, compared with C-2, a large number of three-dimensional network products and voids are observed in the SEM image of II-5. Combined with subsequent EDS analysis, the gelatinous products can be qualitatively analyzed. The EDS result in Figure 8c shows that the geopolymer gel (point c1) is a coexistence composed of N-A-S-H and C-S-H. The main components of the gelatinous product at point c2 are Na, Al, and Si elements, which confirms that the network product is the N-A-S-H gel under a low-calcium system. The increase in the amount of N-A-S-H gel produced is positively correlated with the increase in fly ash content in II-5 [52]. It can be observed from Figure 8a,b that this stable three-dimensional network structure hinders the secondary reaction between the N-A-S-H gel and the hydration product of the slag, which results in a decrease in the mutual adhesion between soil particles [52]. In Figure 8d, there is a small amount of unreacted fly ash around the gelatinous products, and partially exposed soil particles are observed. The above UCS test results show that the UCS of II-5 are 0.79 and 1.15 MPa at 7 d and 28 d, respectively, which is even lower than C-2. This indicates that excessive fly ash will have a detrimental effect on the UCS of stabilized soil [51,52].

## 4. Conclusions and Observations

In this study, the UCS test, scanning electron microscope (SEM), and X-ray energy spectrum analysis (EDS) were used to comparatively investigate the unconfined compressive strength (UCS) and microstructure characteristics of cement stabilized soil and slag and fly ash-based geopolymer stabilized soil. Based on the actual engineering background, an optimal mix-proportions scheme for geopolymer stabilized soil is proposed. The following conclusions can be drawn:

(1) Increasing the stabilizer content and extending the curing period can increase the UCS of stabilized soil. When the stabilizer content increases from 12% to 18%, the UCS of cement stabilized soil increases almost linearly, while the UCS growth rate of geopolymer soil gradually decreases.

(2) The strength of the geopolymer soil with a large proportion of slag develops faster and obtains a higher 28 d UCS. Under the same conditions, the UCS of the geopolymer soil only slightly decreases when the slag–fly ash ratio changes from 90:10 to 80:20, while the UCS decreases significantly when the slag–fly ash ratio changes from 80:20 to 70:30.

(3) The UCS of the geopolymer soil increases first and then decreases with an increase in the alkaline activator content. The maximum value is obtained at an alkaline activator content of 30%.

(4) Different from cement stabilized soil, geopolymer soil rapidly produces a large number of uniformly distributed gelatinous products in the presence of an alkaline activator which bind the soil particles tightly. The EDS results show that the gelatinous products are composed of C-S-H gel and sodium-based aluminosilicate (N-A-S-H). In addition, it is found that the increase in the amount of N-A-S-H gel produced is positively correlated with the increase in fly ash content.

In conclusion, it is recommended to adopt a mix-proportions scheme with 15% of geopolymer content, 80:20 as the slag–fly ash ratio, and 30% of alkaline activator content for geopolymer stabilized riverside soft soil, that is, II-3. The 7 d and 28 d UCS of II-3 are 1.21 and 1.68 MPa, respectively. Its 28 d UCS is 56.0 times that of undisturbed soil and 1.39 times that of cement stabilized soil with the same stabilizer content.

## Figures and Tables

**Table 1:** Physical characteristics of test soil.

Sampling Depth/m	Specific Gravity	Natural Moisture Content/%	Wet Density/g·cm^−3^	Liquid Limit/%	Plasticity Index	Initial Void Ratio	Organic Matter Content/%
6.0	2.65	51.8	1.63	49.4	26.9	1.503	2.21

**Table 2:** Chemical composition of raw materials.

Raw Materials	MgO	Al_2_O_3_	SiO_2_	CaO	Fe_2_O_3_	SO_3_
Cement	3.30	5.50	21.00	65.40	2.90	2.00
Slag	6.01	17.70	34.50	34.00	1.03	1.64
Fly ash	0.86	27.4	49.04	3.23	1.53	1.15

**Table 3:** Composition and basic properties of geopolymer.

Slag:Fly Ash	Alkaline Activator		Water–Binder Ratio	Bleeding Rate/%	Setting Time/Min		Compressive Strength/MPa	
	Modulus	Content/%			Initial	Final	7 d	28 d
90:10	1.2	40	0.5	0.52	153	245	49.4	65.2

**Table 4:** Mix proportions.

Label	Cement Content/%	Geopolymer Content/%	Slag:Fly Ash	Alkaline Activator		Water–Binder Ratio
				Modulus	Content/%	
C-1	12	-	-	1.2	-	0.5
C-2	15	-	-	1.2	-	0.5
C-3	18	-	-	1.2	-	0.5
I-1	-	12	90:10	1.2	40	0.5
I-2	-	12	80:20	1.2	40	0.5
I-3	-	12	80:20	1.2	30	0.5
I-4	-	12	80:20	1.2	20	0.5
I-5	-	12	70:30	1.2	20	0.5
II-1	-	15	90:10	1.2	40	0.5
II-2	-	15	80:20	1.2	40	0.5
II-3	-	15	80:20	1.2	30	0.5
II-4	-	15	80:20	1.2	20	0.5
II-5	-	15	70:30	1.2	20	0.5
III-1	-	18	90:10	1.2	40	0.5
III-2	-	18	80:20	1.2	40	0.5
III-3	-	18	80:20	1.2	30	0.5
III-4	-	18	80:20	1.2	20	0.5
III-5	-	18	70:30	1.2	20	0.5

## Data Availability

The data presented in this study are available on request from the corresponding author.
